# Dissecting recipient from donor contribution in experimental kidney transplantation: focus on endothelial proliferation and inflammation

**DOI:** 10.1242/dmm.035030

**Published:** 2018-07-17

**Authors:** Diana A. Papazova, Merle M. Krebber, Nynke R. Oosterhuis, Hendrik Gremmels, Arjan D. van Zuilen, Jaap A. Joles, Marianne C. Verhaar

**Affiliations:** 1 Department of Nephrology and Hypertension, UMC Utrecht, POB 85500, 3508 GA Utrecht, The Netherlands; 2 Department of Anesthesiology, Amsterdam UMC, Vrije Universiteit Amsterdam, POB 7057, 1007 MB Amsterdam, The Netherlands

**Keywords:** Kidney transplantation, Chronic kidney disease, Glomerular endothelium, Circulating cells, Inflammation

## Abstract

Kidney transplantation (Tx) is considered the only definite treatment for end-stage kidney disease (ESKD) patients. The increasing prevalence of ESKD has necessitated the introduction of transplantation with kidneys from suboptimal donors. There is, however, still a lack of fundamental and longitudinal research on suboptimal kidney transplants. Specifically, there is a demand for accurate pre-Tx predictors of donor kidney function and injury to predict post-Tx outcome. In the present study, we combine rat models of chronic kidney disease (CKD) and renal Tx to dissect the effects of healthy and CKD renal grafts on healthy and CKD recipients. We show that renal function at 6 weeks post-Tx is exclusively determined by donor graft quality. Using cell tracking within enhanced green fluorescent protein-positive (eGFP^+^) recipients, we furthermore show that most inflammatory cells within the donor kidney originate from the donor. Oxidative and vascular extra-renal damage were, in contrast, determined by the recipient. Post- versus pre-Tx evaluation of grafts showed an increase in glomerular and peritubular capillary rarefaction in healthy but not CKD grafts within a CKD environment. Proliferation of glomerular endothelium was similar in all groups, and influx of eGFP^+^ recipient-derived cells occurred irrespective of graft or recipient status. Glomerular and peritubular capillary rarefaction, severity of inflammation and macrophage subtype data post-Tx were, however, determined by more complicated effects, warranting further study. Our experimental model could help to further distinguish graft from recipient environment effects, leading to new strategies to improve graft survival of suboptimal Tx kidneys.

This article has an associated First Person interview with the first author of the paper.

## Abstract (precis)

**Summary:** Using experimental kidney transplantation, we dissected donor graft from recipient environment effects, focusing on the endothelium and inflammation. These results can direct strategies to improve graft survival after suboptimal transplantation.

## INTRODUCTION

Kidney transplantation (Tx) is considered the only definite treatment option for patients with end-stage kidney disease (ESKD). Graft function and survival in kidney transplant patients have improved over time, especially by limiting acute and late acute rejection events (Heeman and Lutz, 2013). However, graft function still deteriorates owing to time-dependent immunologic and nonimmunologic causes (Brouard et al., 2011; Nankivell et al., 2003). The quality of the donor kidney at the time of Tx [influenced by age, hypertension and decreased glomerular filtration rate (GFR)] is an important determinant of transplant outcome (Legendre et al., 2013). Decreasing supply of donors, coupled with increasing recipient demand, has led to the necessity for using suboptimal kidney donors. Fundamental and longitudinal research on transplanting suboptimal kidneys, including accurate pre-Tx predictors of donor kidney function and injury in relation to post-Tx outcome, is not yet available. Besides the condition of the donor kidney, patient characteristics (age, comorbidities) of the recipient are important for graft function, morphology and long-term graft function and structure (Legendre et al., 2013). Chronic kidney disease (CKD) is characterized by oxidative stress, a pro-inflammatory state, endothelial dysfunction and uremic toxins (Oberg et al., 2004; Stinghen and Pecoits-Filho, 2011), and reduced renal regeneration (Jie et al., 2010; Kelley et al., 2010; Klinkhammer et al., 2014; Peired et al., 2013; van Koppen et al., 2012b; Yamada et al., 2014). All these factors contribute to the progression of CKD and, at the time of Tx, could result in a systemic environment detrimental to the graft.

Isogenic experimental Tx allows researchers to dissect the influence of the CKD recipient environment from graft function in an immunocompetent context. The symmetrical bilateral ablation (BA) rat model we introduced (Papazova et al., 2014) can be used in Tx studies for developing strategies to improve graft survival after Tx of suboptimal kidneys. It allows us to compare injury of optimal and suboptimal donor grafts pre- and post-Tx in healthy and CKD systemic environments. It could also help to dissect the complex crosstalk between graft and recipient tissues in an isogenic setting lacking immunosuppression and low-grade rejection, in particular the influx of recipient-derived cells. Previous studies suggest incorporation of bone marrow-derived endothelial progenitor cells in renal grafts, but the data are conflicting (Kwon et al., 2010). We have previously reported that injections of bone marrow cells in the renal artery protected against glomerular damage in experimental CKD, despite the lack of incorporation or transdifferentiation of these cells into the glomerular endothelium (van Koppen et al., 2012a).

The primary aims of this study were to establish differences in graft function/structure in general, with a particular focus on glomerular and peritubular capillary rarefaction and the presence of inflammation in three outcomes: function of suboptimal (CKD) versus optimal (healthy) donor grafts in CKD versus healthy systemic environments (cross-Tx), structure of post-cross-Tx grafts versus pre-cross-Tx biopsies, and effects of recipient-derived cells in cross-Tx. Based on previous observations, we had three hypotheses: (1) long-term graft function and injury are determined by interaction between graft quality and recipient environment at the time of Tx, (2) suboptimal donors lead to further decrease in graft function and injury post-Tx, and (3) incorporation of recipient-derived cells is not determined by the status of the recipient environment.

To evaluate the contribution of recipient cells to damage in renal grafts, enhanced green fluorescent protein-positive (eGFP^+^) Lewis rats (van den Brandt et al., 2004; van Koppen et al., 2012a) were used as recipients in our model of experimental renal Tx.

## RESULTS

### Experimental groups

**Fig. 1.**
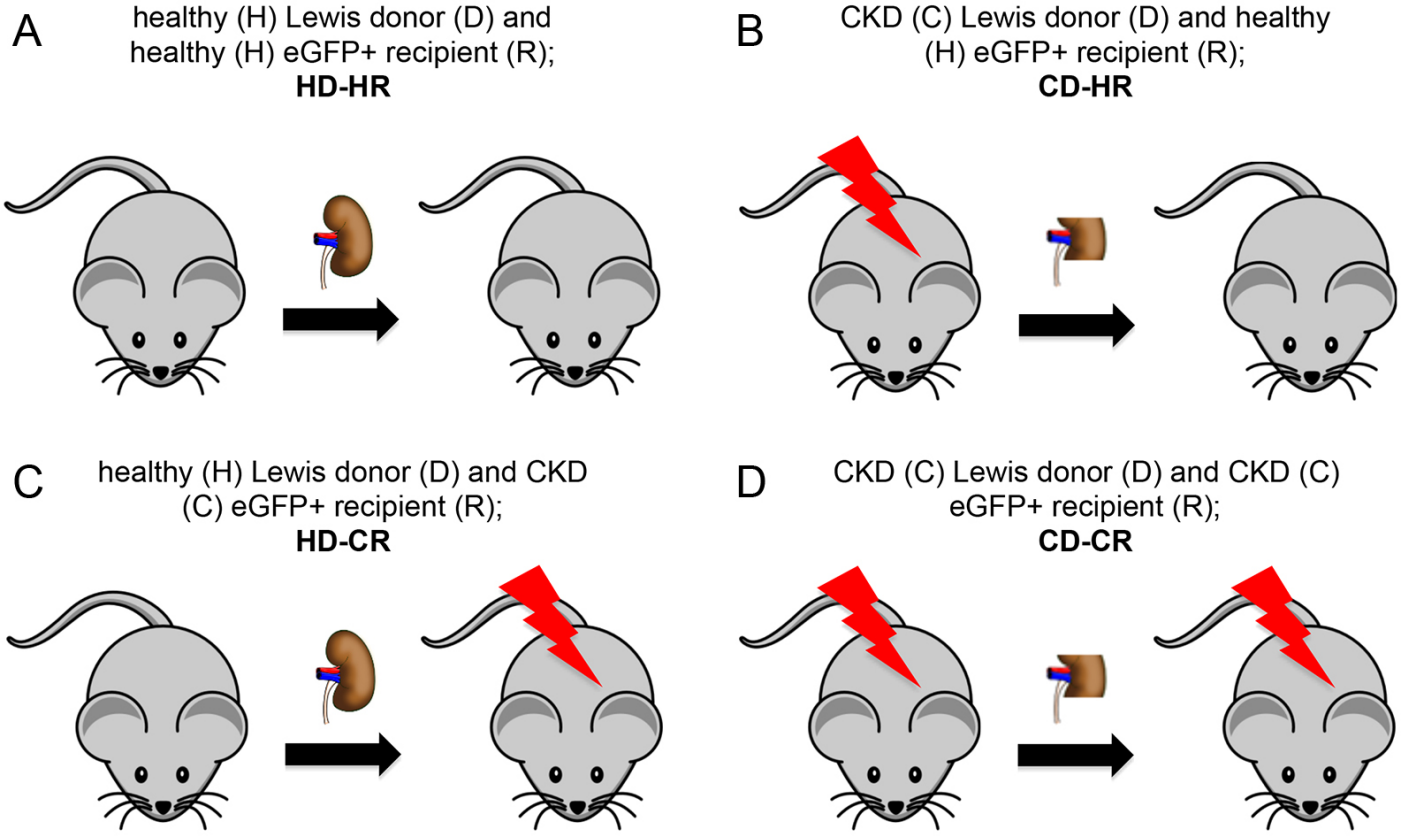
**Experimental set-up.** The following groups were used: (A) HD-HR, healthy (H) Lewis donor (D) and healthy (H) eGFP^+^ recipient (R); (B) CD-HR, CKD (C) Lewis donor (D) and healthy (H) eGFP^+^ recipient (R); (C) HD-CR, healthy (H) Lewis donor (D) and CKD (C) eGFP^+^ recipient (R); (D) CD-CR, CKD (C) Lewis donor (D) and CKD (C) eGFP^+^ recipient (R) (*n*=6/group). Body weights and ages of all rats at relevant stages of the experiment are shown in Tables S1 and S2.

Our experimental cross-Tx design was as follows: HD-HR, healthy (H) Lewis donor (D) and healthy (H) eGFP^+^ recipient (R); CD-HR, CKD (C) Lewis donor (D) and healthy (H) eGFP^+^ recipient (R); HD-CR, healthy (H) Lewis donor (D) and CKD (C) eGFP^+^ recipient (R); CD-CR, CKD (C) Lewis donor (D) and CKD (C) eGFP^+^ recipient (R) (*n*=6/group) (Fig. 1; Tables S1 and S2).

### Pre-transplantation data in donors and recipients

At week 1 (baseline) before donation/Tx, all rats (*n*=24) that underwent bilateral ablation (BA), on average, had higher systolic blood pressure (SBP) (136±21 versus 110±15 mmHg, *P*<0.001), proteinuria (111±31 versus 10±3 mg/d, *P*<0.001) and plasma urea (9.9±1.8 versus 5.8±0.8 mmol/l, *P*<0.001) than all control rats (*n*=24). Pre-Tx data for all four donor and recipient combinations ∼1 week prior to surgery are shown in Table S3.

### Longitudinal data

Increased body weight was observed in the CKD recipients (CR) compared with the healthy recipients (HR), corresponding to the age difference (*P*<0.05) in our cross-Tx set-up. The SBP of the recipient was influenced by the transplanted kidney at week 5 (Table 1; Fig. S1): SBP in the CR was lower with a healthy donor (HD) than with a CKD donor (CD). The HR had a higher SBP with a CD than with an HD (both *P*<0.05). A comparable pattern was observed for urea and proteinuria. HD-CR rats had lower urea and proteinuria compared with CD-CR, but higher proteinuria compared with HD-HR, at weeks 3 and 5 (all *P*<0.05). CD-HR rats had higher urea and proteinuria compared with HD-HR and CD-CR at weeks 3 and 5 (all *P*<0.05). The lower urea in the CD-CR rats could have been caused by less chow intake than the CD-HR rats, but this was not recorded.

**Table 1.**
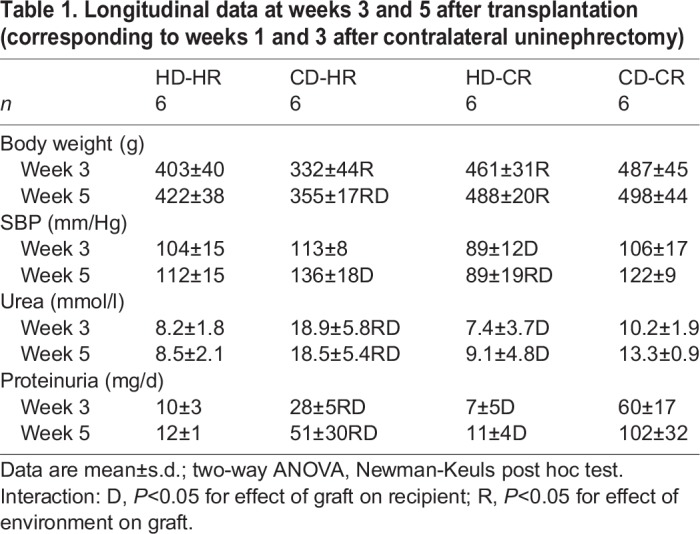
**Longitudinal data at weeks 3 and 5 after transplantation (corresponding to weeks 1 and 3 after contralateral uninephrectomy)**

### Terminal data

Terminal renal function data were not available for one rat (in the CD-CR group) that died, possibly of heart failure, during the terminal experiment. This rat had no cardiac output immediately after being anesthetized and could not be resuscitated. Postmortem, we observed a markedly dilated heart with multiple fibrotic scars in the myocardium.

### Renal function

Anemia (decreased hematocrit) and decreased renal function (increased plasma urea and decreased GFR measured by inulin clearance) at termination confirmed marked impairment after ablation. This was exclusively determined by donor status: recipient environment did not influence outcome [CD-HR versus CD-CR and HD-CR versus HD-HR; comparisons for all parameters were nonsignificant (NS)] (Table 2; Fig. S2). A comparable pattern was observed for renal hemodynamics (RPF, renal plasma flow; RBF, renal blood flow; RVR, renal vascular resistance) and fractional excretions of sodium and potassium (FE_Na_ and FE_K_, respectively).

**Table 2.**
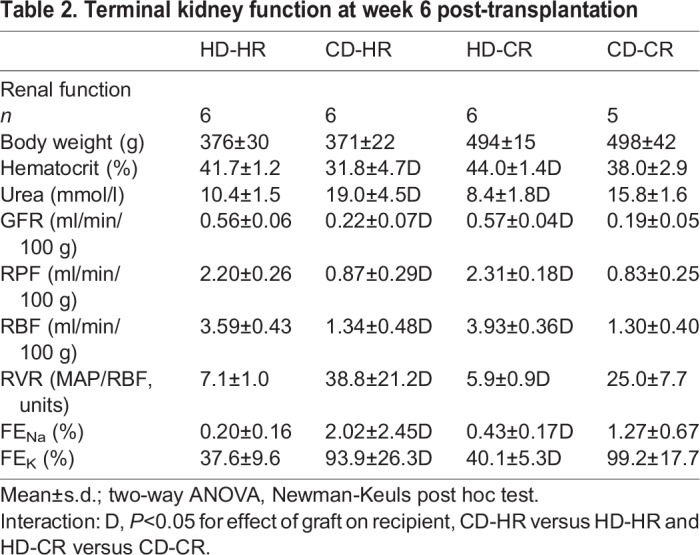
**Terminal kidney function at week 6 post-transplantation**

### Systemic damage

**Fig. 2.**
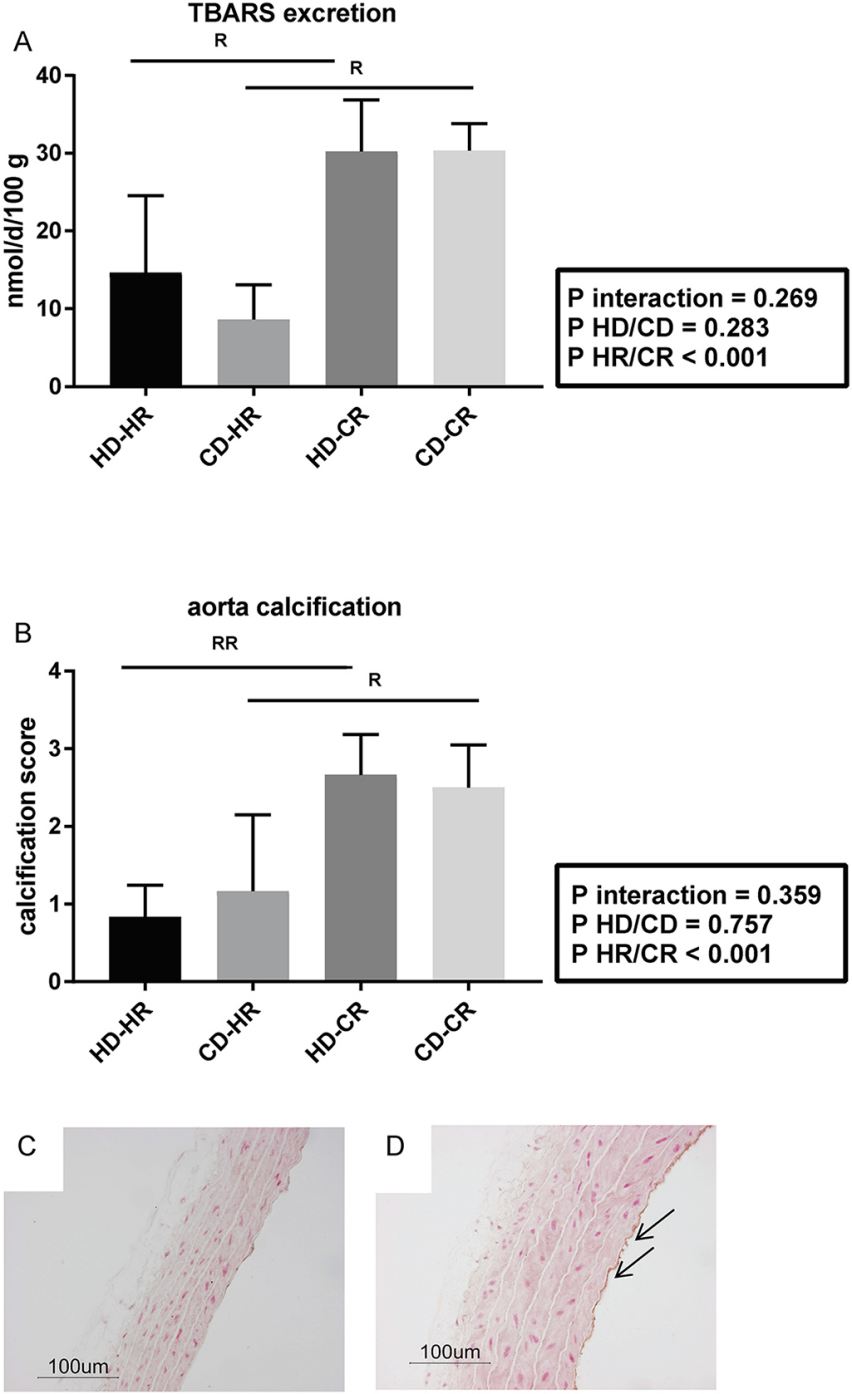
**Systemic oxidative damage and vascular damage.** (A) TBARS excretion. (B) Aorta calcification (von Kossa stain). HD, healthy donor; CD, donor with CKD (pre-Tx); HR, healthy recipient; CR, recipient with CKD (post-Tx). All *n*=6. Data are mean±s.d.; two-way ANOVA, Newman-Keuls post hoc test. R, *P*<0.05; RR, *P*<0.01. Post-transplantation CR versus HR, *P*<0.05 for both variables (not shown in graphs). (C,D) Representative fixed paraffin-embedded histology post-transplantation of HD-HR (C) and HD-CR (D) combinations. Arrows indicate subendothelial calcification of aortas.

Thiobarbituric acid reactive substance (TBARS) excretion was exclusively determined by recipient environment and was higher in the CR versus HR, irrespective of the transplanted kidney (*P*<0.05 for CR versus HR; Fig. 2A). A similar pattern was observed for vascular damage assessed by scoring aorta calcification (*P*<0.05 for CR versus HR; Fig. 2B-D). Note that with the low phosphate content of the standard rodent diet used in this study (0.63% w:w), calcification is restricted to the subendothelium.

### Renal damage

**Fig. 3.**
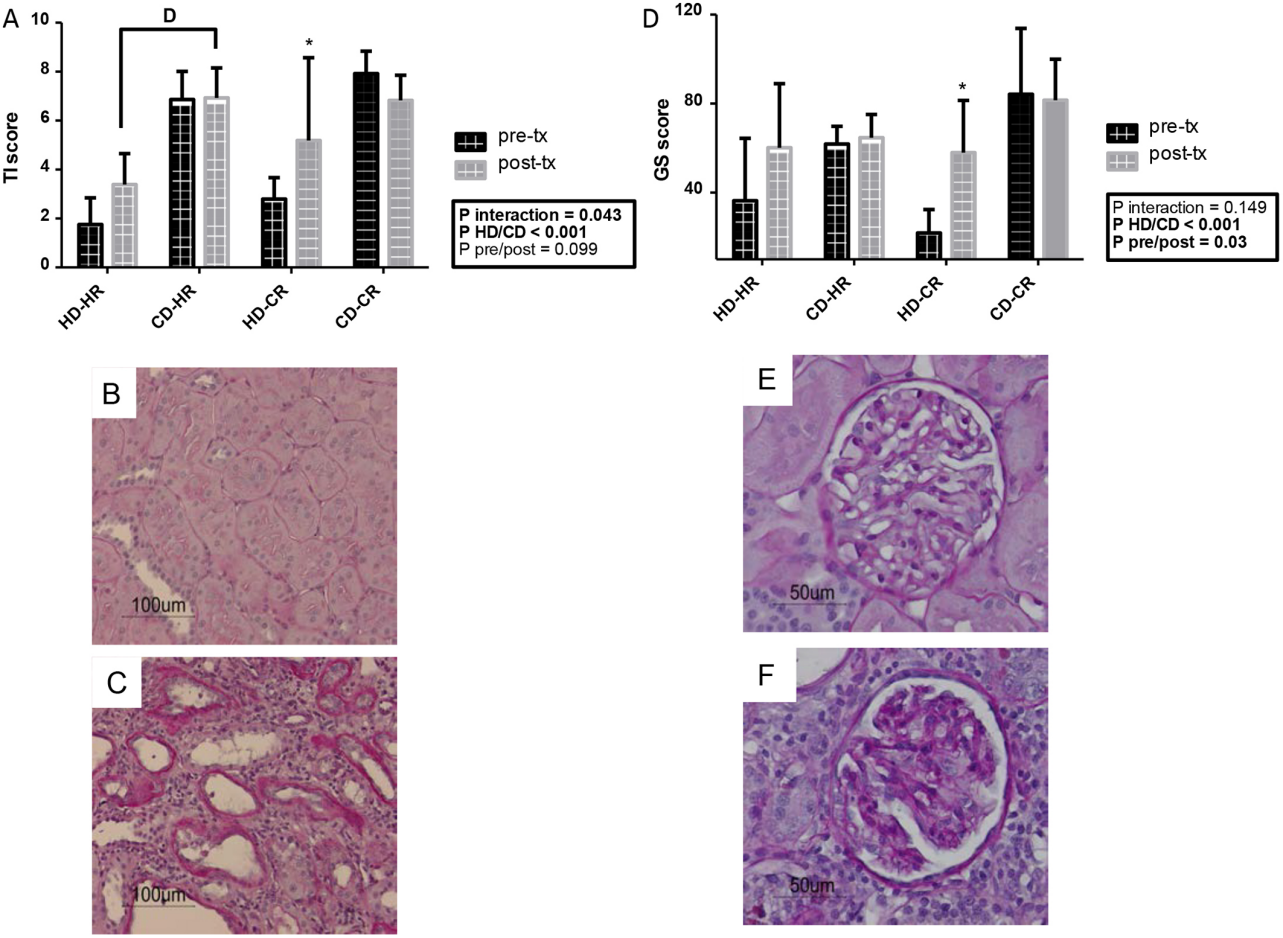
**Histology pre- and post-transplantation (PAS staining).** (A) TI score. (D) GS score. (B,C,E,F) Representative fixed paraffin-embedded histology pre- (B,E) and post-transplantation (C,F) of an HD-CR combination (analogous to clinical transplantation with a living donor) for TI (B,C) and GS (E,F). HD, healthy donor; CD, donor with CKD (pre-Tx); HR, healthy recipient; CR, recipient with CKD (post-Tx). All *n*=6. Data are mean±s.d.; two-way repeated measures ANOVA, Newman-Keuls post hoc test. *P<0.05 versus pre-transplantation. Interaction: D, *P*<0.05 for effect of graft on recipient. Pre-transplantation CD versus HD, *P*<0.05 for all variables (post hoc symbols not shown in graphs).

**Fig. 4.**
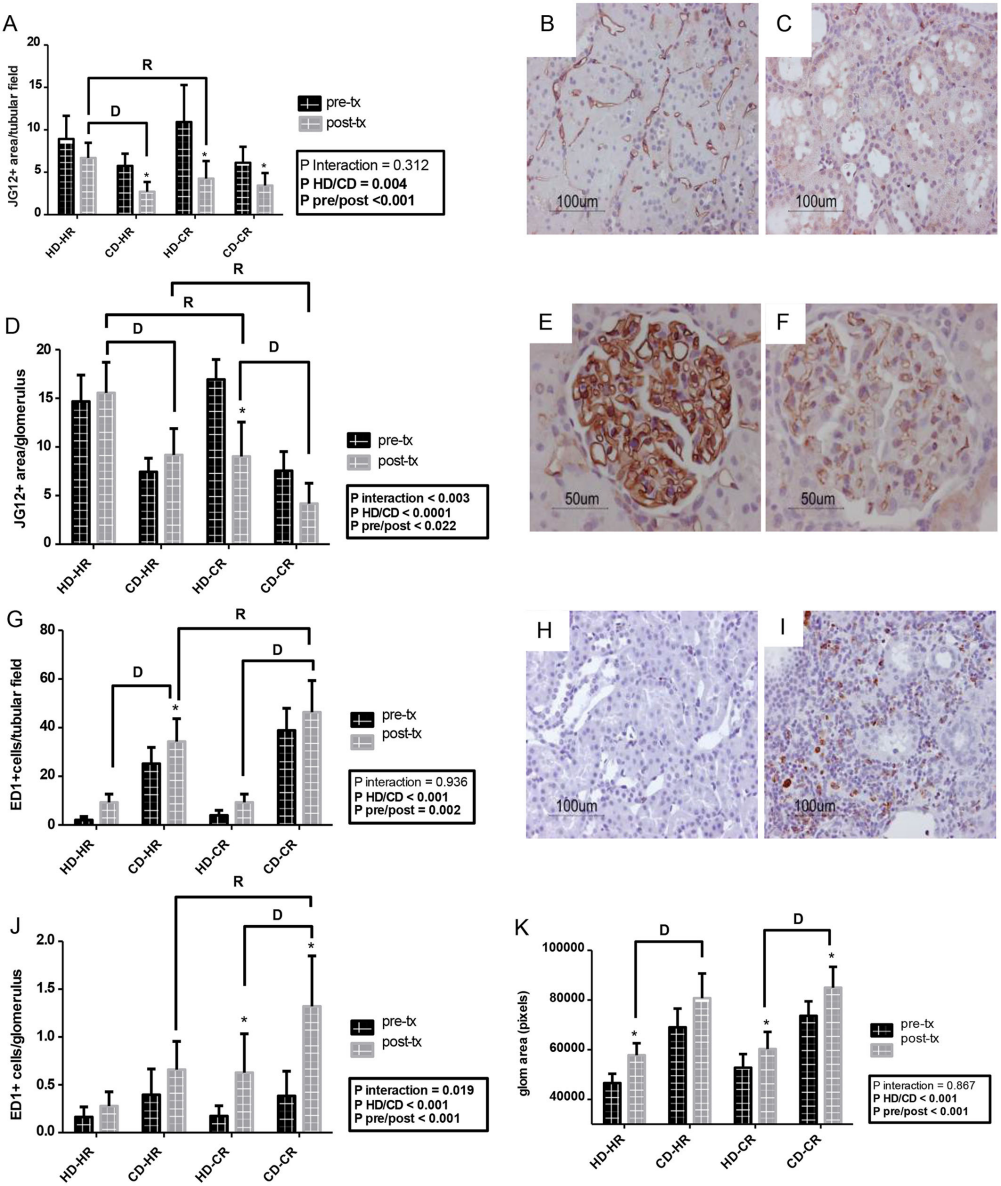
**Immunohistochemistry of pre- and post-transplantation kidneys.** (A) JG12^+^ area/tubular field, (D) JG12^+^ area/glomerulus, (G) ED1^+^ cells/tubular field, (J) ED1^+^ cells/glomerulus and (K) glomerular area (scored on slides stained with JG12). (B,C,E,F,H,I) Representative fixed paraffin-embedded histology pre- (B,E,H) and post- (C,F,I) transplantation of an HD-CR combination (analogous to clinical transplantation with a living donor). HD, healthy donor; CD, donor with CKD (pre-Tx); HR, healthy recipient; CR, recipient with CKD (post-Tx). All *n*=6. Data are mean±s.d.; two-way repeated measures ANOVA, Newman-Keuls post hoc test. *P<0.05 versus pre-transplantation. Interaction: D, *P*<0.05 for effect of graft on recipient; R, *P*<0.05 for effect of environment on graft. Pre-transplantation CD versus HD, *P*<0.05 for all variables (post hoc symbols not shown in graphs).

Representative histology [periodic acid Schiff (PAS) staining] and immunohistochemistry [aminopeptidase P (JG12) and ED1 (also known as EDA)] pre- and post-Tx of a HD-CR combination (analogous to clinical transplantation with a living donor graft) is shown in Figs 3 and 4. Comparing donors' reference kidneys pre-Tx, we observed marked differences between the CD and HD for all histological parameters (two-way repeated measures ANOVA, HD/CD *P*<0.01; Figs 3 and 4). Comparison of tubulo-interstitial injury (TI) and focal glomerulosclerosis (GS) post- versus pre-Tx (Fig. 3) clearly showed aggravation of injury in HD-CR in both parameters (Fig. 3A,D), while a CKD donor graft in the healthy recipient environment (CD-HR) resulted in halted injury progression, and a CKD donor graft in a CKD recipient (CD-CR) did not result in further deterioration. TI showed significant post-Tx differences between HD and CD in the HR but not in the CR, probably due to large variation in the HD-CR data (Fig. 3A-C). GS scores in HD versus CD injury in all recipients were also not significantly different, whereas GS increased in the HD-CR but remained stable in the CD-CR (Fig. 3D-F; individual data in Fig. S3).

### Renal endothelium

Comparisons of renal peritubular capillary and glomerular rarefaction (JG12^+^) post- versus pre-Tx, showed marked decreases in HD-CR (Fig. 4A-F) endothelial area in both peritubular capillaries and glomeruli. Note that in glomeruli, no other post- versus pre-Tx changes occurred, whereas in tubular fields, only HD-HR remained stable. Glomerular area was consistently increased in post-Tx versus pre-Tx kidneys and in CD versus HD kidneys (Fig. 4K). Post-Tx, fewer peritubular capillaries and glomerular endothelium were observed in healthy donor grafts transplanted in the CKD recipient environment than in the healthy recipient environment (Fig. 4A,D, *P*<0.05), and in CKD versus healthy donor grafts in a healthy recipient environment (Fig. 4A,D, *P*<0.05). Donor and recipient environment effects on endothelium were more pronounced in glomeruli compared with peritubular capillaries. The strongest donor-recipient interaction effect was observed for glomerular endothelial area relative to glomerular area (Fig. 4D, *P*<0.003; representative histology in Fig. 4E,F; individual data in Fig. S6). Note that the glomerular area size distribution within CD kidneys was much broader than in HD kidneys (Fig. S4).

### Renal inflammation and macrophage subtype

Signs of inflammation (ED1^+^) were significantly more evident in post- versus pre-Tx in CD-HR tubular fields (Fig. 4G-I) and HD-CR and CD-CR glomeruli (Fig. 4J). At 6 weeks post-Tx, we observed fewer ED1^+^ cells per tubular field and glomerulus in CD-HR compared with CD-CR (Fig. 4G,J, *P*<0.05) and in HD-CR compared with CD-CR (Fig. 4G,J, *P*<0.05). ED1 inflammation was primarily determined by donor status in tubular fields and by donor and donor-recipient interaction in glomeruli.

**Fig. 5.**
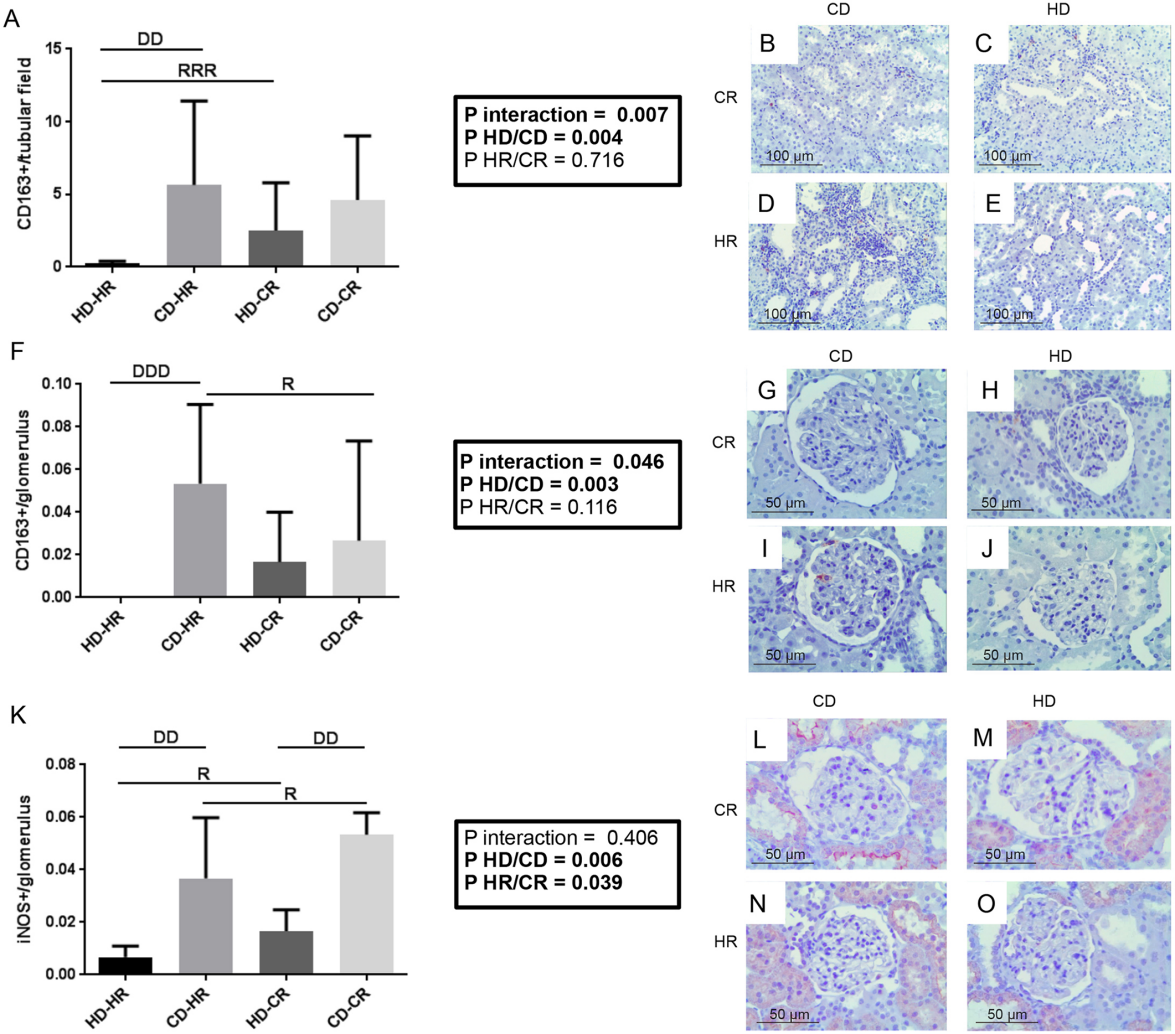
**Immunohistochemistry of CD163 and iNOS inflammation post-transplantation at week 6.** (A-O) Representative fixed paraffin-embedded histology with CD163^+^ (M2 MQs) cells per tubular field (A-E) and per glomerulus (F-J), and iNOS^+^ (M1 MQs) cells per glomerulus (K-O). All *n*=6. Data are mean±s.d.; two-way ANOVA, Newman-Keuls post hoc test. Interaction: D, *P*<0.05, DD, *P*<0.01, DDD, *P*<0.001 for effect of graft on recipient; R, *P*<0.05, RRR, *P*<0.001 for effect of recipient environment on graft.

Next, we used inducible nitric oxide synthase (iNOS) and CD163 (also known as M130) immunohistochemistry to determine relative contributions of M1 and M2 macrophages (MQs) post-Tx. For CD163, an M2 marker, CD versus HD in a HR increased the number of positive cells per glomerulus and tubular field (Fig. 5A-J, *P*<0.01 and *P*<0.001, respectively). HD-CR also had significantly increased tubular expression of CD163 compared with HD-HR (Fig. 5A, *P*<0.001). For iNOS, and M1 marker, both CD versus HD and CR versus HR increased glomerular expression. Here, either donor or recipient environment status, but not donor-recipient interaction, determined the outcome (Fig. 5K-O). The increased expression of iNOS and CD163 in CD compared with HD is in agreement with increased ED1^+^ cells. However, although iNOS expression was highest in CD-CR, CD163 expression was highest in CD-HR. Donor and donor-recipient interaction effects determined the percentage of CD163^+^ MQs as part of the ED1 population in glomeruli, while there was no difference in iNOS. In tubular fields, donor-recipient interaction determined CD163^+^ and remaining ED1^+^ percentage (Fig. S5A,B).

### Presence of recipient-derived eGFP^+^ cells and endothelial proliferation

**Fig. 6.**
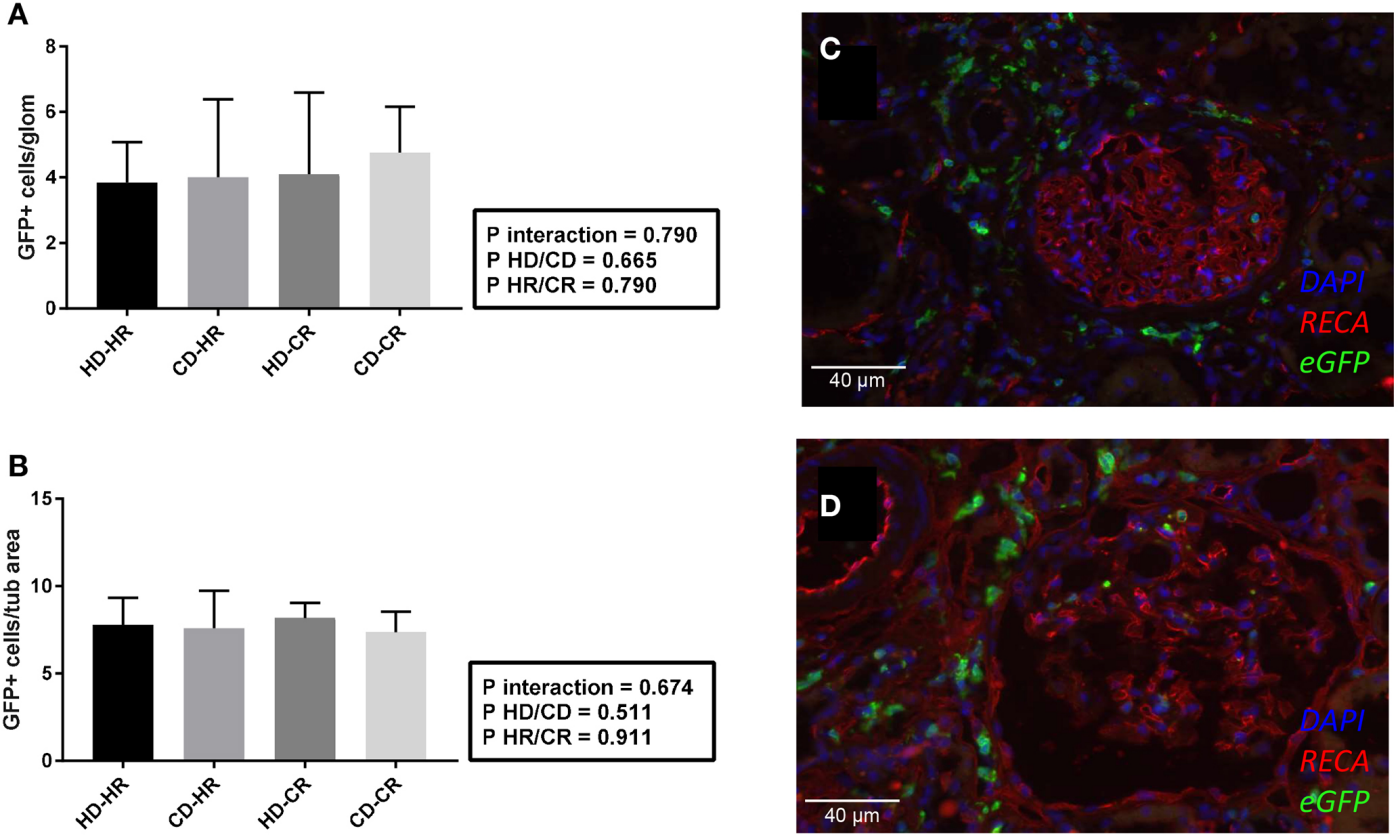
**Immunofluorescent staining post-transplantation at week 6.** (A) eGFP^+^ cells/glomerulus. (B) eGFP^+^ cells/tubular area. (C,D) Representative snap-frozen immunofluorescent staining of HD-CR (C) and CD-CR (D) combinations. RECA (red), 4′,6-diamidino-2-phenylindole (DAPI, blue) and eGFP green.

**Fig. 7.**
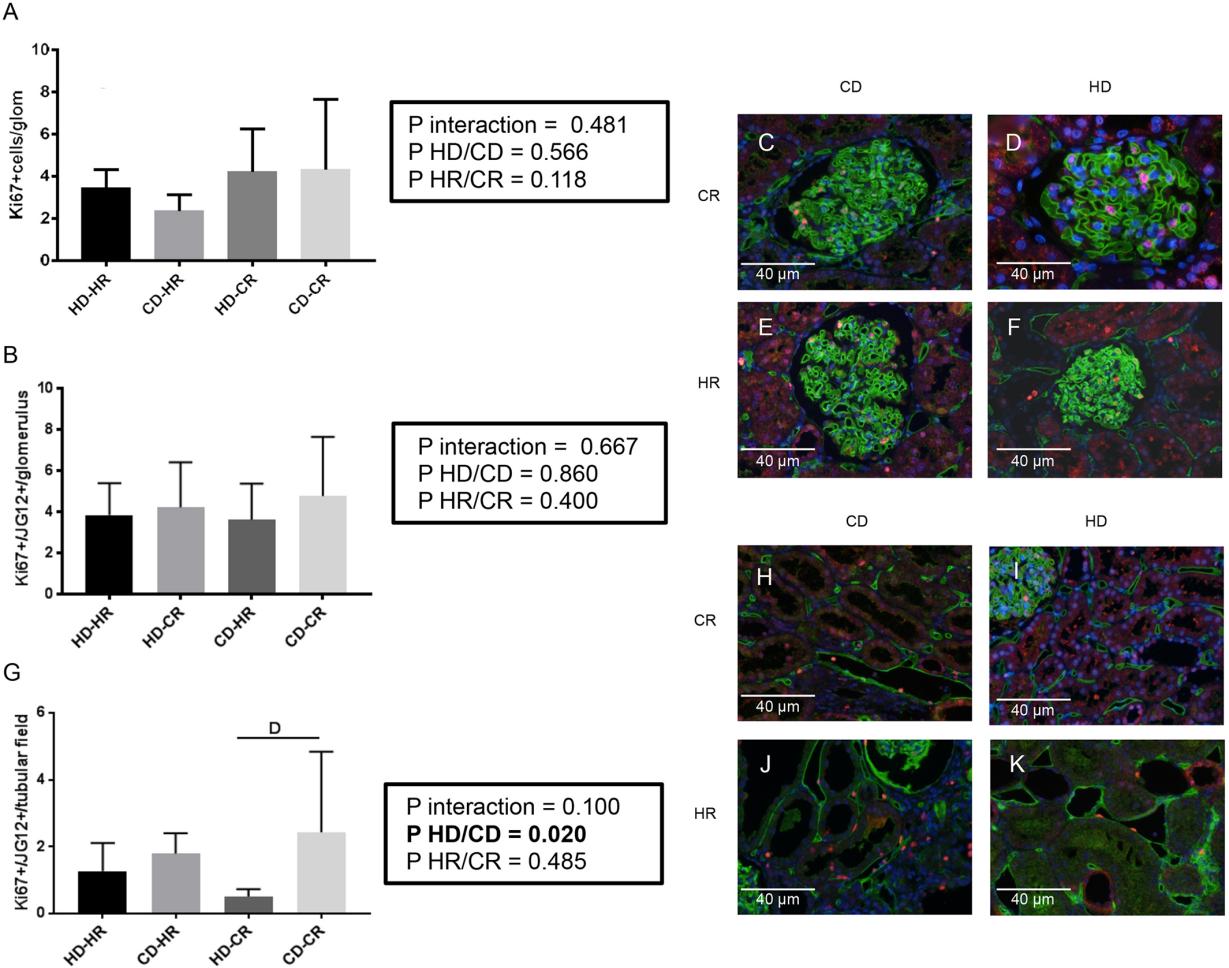
**Immunofluorescent staining post-transplantation at week 6.** (A-K) Ki67 (green, A), JG12 (tyramide FITC, green) and Ki67 (tyramide TRITC, red) immunofluorescence. DAPI, blue. Representative fixed paraffin-embedded histology with JG12^+^/Ki67^+^ cells per glomerulus (B-F) and per tubular field (G-K). All *n*=6. Data are mean±s.d., two-way ANOVA, Newman-Keuls post hoc test. Interaction: D, *P*<0.05 for effect of graft on recipient.

To address the contribution of recipient cells in progression of damage, peritubular and glomerular capillary rarefaction or inflammation post-Tx, we performed eGFP^+^/rat endothelial cell antigen (RECA) immunofluorescent staining. No significant differences were found in kidneys of the recipients for eGFP^+^ cells/glomerulus or eGFP^+^ area/tubular field (Fig. 6A-D). To examine whether endothelial proliferation post-Tx differed between experimental groups, Ki67 (also known as Mki67) staining and JG12/Ki67 double-immunofluorescent staining were performed. No significant differences were found in proliferating cells expressing Ki67 in the glomeruli of post-Tx kidneys (Fig. 7A)*.* Peritubular expression of JG12/Ki67 (Fig. 7B-F) was determined by donor status (*P*<0.05) and was higher in the CKD donors than in healthy donors in the CR. However, glomerular JG12^+^/Ki67^+^ (Fig. 7G-K) was similar in all groups (NS).

### Recipient bone marrow-derived inflammatory cells

**Fig. 8.**
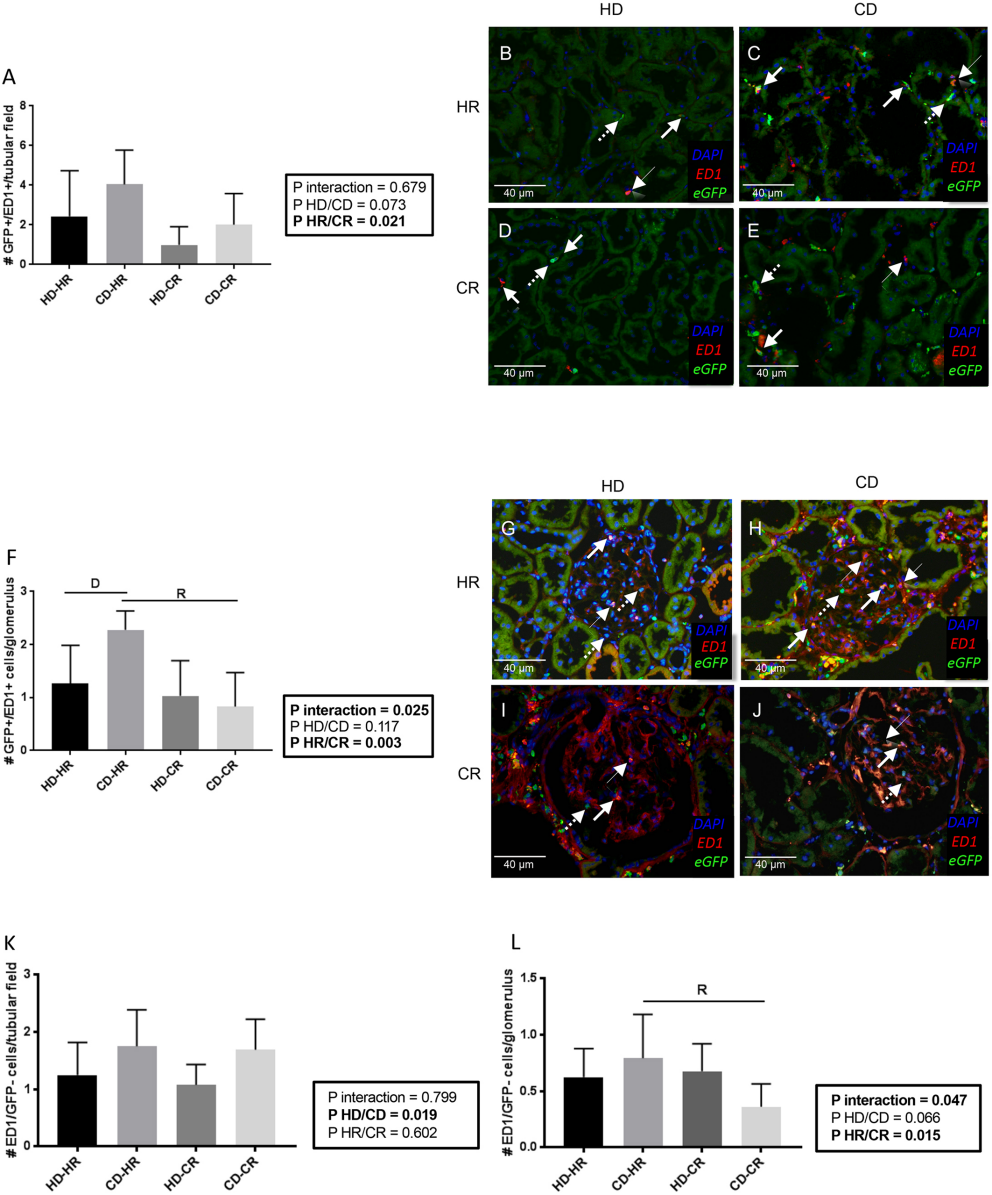
**Immunofluorescent staining post-transplantation at week 6.** (A-J) Representative snap-frozen histology of tubular fields (A-E) and glomeruli (F-J). DAPI (blue), ED1 (CD68, red) and eGFP (green). Representative positive cells are depicted as follows: solid-line arrows indicate ED1^+^/eGFP^+^ cells, arrowheads indicate ED1^+^/eGFP^−^ cells and dotted-line arrows indicate ED1^−^/eGFP^+^ cells. (K,L) Number of eGFP^−^/ED1^+^ cells per tubular field (K) or per glomerulus (L). All *n*=6. Data are mean±s.d.; two-way ANOVA, Newman-Keuls post hoc test. Interaction: D, *P*<0.05 for effect of graft on recipient; R, *P*<0.05 for effect of recipient environment on graft.

Using eGFP/ED1 double staining, the relative contribution of recipient environment and graft to eGFP^+^ inflammatory cells was examined. The number of eGFP^+^/ED1^+^ cells per tubular field was determined by the recipient environment (Fig. 8A-E). In glomeruli, there was a donor-recipient interaction effect: CD increased eGFP^+^/ED1^+^ cells in the HR compared with the CR, and compared with the HD in the HR (Fig. 8F-J, *P*<0,05). The presence of eGFP^−^/ED1^+^ cells per tubular field was exclusively determined by donor status (Fig. 8K, *P*=0.019), being higher in CD independent of recipient status. The number of eGFP^−^/ED1^+^ cells per glomerulus was influenced by donor-recipient interaction (Fig. 8L), although most ED1^+^ cells were donor derived. No differences were found between groups in the proportion of ED1^+^ cells as part of the eGFP^+^ population [tubulo-interstitial (19±10%, *n*=24) and glomerular (36±16%, *n*=24)] or vice-versa [tubulo-interstitial (18±12%, *n*=24) and glomerular (22±12%, *n*=24)] (Fig. S8C-F).

## DISCUSSION

The primary aim of this study was to establish differences in graft function and structure, with particular attention to peritubular and glomerular capillary rarefaction and inflammation. This could only be studied in the context of our CKD/Tx model that combines two unique features (Papazova et al., 2014): symmetrical injury in CKD donors allowing a baseline ‘biopsy’ kidney, and eGFP^+^ recipients in combination with cross-transplantation, allowing identification of impact of recipient environment. Using this model, we could investigate three different conditions: (1) comparing optimal (healthy) versus suboptimal (CKD) donor grafts in healthy and CKD systemic environments (cross-Tx), (2) comparing post-cross-Tx grafts versus pre-cross-Tx biopsies, and (3) exploring the effects of recipient-derived cells in cross-Tx. Our study was descriptive in the sense that there was no donor and/or recipient treatment prior to surgery.

### Condition 1: comparing optimal (healthy) and suboptimal (CKD) donor grafts in healthy and CKD systemic environments (cross-Tx)

For the first time, our symmetrical study design shows that post-Tx graft function is solely determined by donor status. Despite significant improvement of SBP, proteinuria and urea when comparing healthy with CKD donors in CKD recipients, recipient environment or interaction effects between recipient and donor did not affect renal function. Suboptimal donors in both healthy and CKD recipient environments had significantly lower kidney clearance function – as measured by GFR – than healthy donors. Donor status also exclusively influenced progression of GS, and increased peritubular capillary rarefaction and injury of general ED1^+^ inflammation in the tubulo-interstitium.

Clustering of vascular calcification, oxidative stress and endothelial dysfunction is characteristic of CKD and might contribute to enhanced mortality (Kumar et al., 2014). Strikingly, the status of the grafts (healthy versus CKD derived) did not influence systemic oxidative and vascular damage in CKD recipients; it was exclusively determined by recipient environment status. The few patient studies investigating vascular calcification after Tx also do not appear to find calcification regression, with some even noting increases (D'Marco et al., 2015). Although it was previously believed that these results were hampered by immunosuppression, our results suggest that this might not be the case. The increased aortic calcification and oxidative injury in our CKD recipients could have influenced the progression of TI and GS post-Tx (Schiffrin et al., 2007). Indeed, our study shows that both glomerular and peritubular endothelium are damaged when a healthy kidney is transplanted into a CKD rat in comparison to Tx in a healthy recipient (HD-CR versus HD-HR). It is, however, important to note that scarring in rats occurs very quickly, and thus the differences in post-Tx TI and GS scores are probably caused by variation in rate of fibrosis formation rather than regression.

Further characterization of inflammation also found recipient effects on glomerular and tubulo-interstitial macrophage subtype numbers. In human transplant grafts, CD68 count was found to be similar in the presence of interstitial fibrosis/tubular atrophy (IFTA) compared with normal histology (no rejection or IFTA) after Tx (Bergler et al., 2016). The number of CD68^+^ (also known as GP110^+^) infiltrating MQs post-Tx did, however, correlate with the severity of acute rejection, renal function (decline in eGFR) and long-term allograft function (Toki et al., 2014; Bergler et al., 2016; Bräsen et al., 2017). Within the time-span of our experiment, differences in glomerular capillary rarefaction and inflammatory numbers did not associate with corresponding changes in renal function, as donor pre-existent renal damage determined proteinuria and terminal renal function. Clearly, the ablated kidney used as a graft in our experimental study was far more severely injured than the kidneys from living human donors with a subnormal GFR. Nevertheless, in our study, CKD environment persistently determined systemic vascular injury and this might have affected intrarenal glomerular capillary rarefaction to some extent.

MQs are recognized as key players in renal fibrosis and chronic inflammation (Pan et al., 2015; Guiteras et al., 2016). Because MQs primarily arise from bone marrow-derived monocytes, their presence and phenotype might also play a role in vascular injury, repair and regeneration. Generally, MQs can be broadly divided into classically activated (M1) and alternatively activated (M2) phenotypes. M1 MQs are thought to mediate the inflammatory process at the onset of ischemic injury, whereas M2 MQs are involved in postinjury resolution (Salehi and Reed, 2015). By staining for well-established markers, we distinguished M1 (iNOS) and M2 (CD163) MQs (Novak and Koh, 2013; Rubio-Navarro et al., 2016). At 6 weeks post-Tx, the CD163^+^ subpopulation of ED1^+^ cells was highest in the CD-HR. This trend was associated with lack of TI and GS progression, as well as preservation of glomerular and peritubular endothelial area compared with reference kidneys. At this time point, presence of CD163^+^ MQs could promote anti-inflammation and healing, preventing further tissue damage. The presence of interstitial M2 MQs (CD163^+^) in kidney allograft protocol biopsies was previously correlated with both interstitial fibrosis and kidney function, thereby introducing an important pro-fibrotic role for these cells in persistent lesions (Ikezumi et al., 2015; Braga et al., 2015). Complete understanding of M2 phenotype function in the setting of chronic rejection still requires additional research. Despite the utility of the M1/M2 paradigm for analysis, we should note that the biological reality is more complex. In a single cell *in vivo*, these markers can also be co-expressed or not expressed at all. This could explain the high percentage of ED1^+^ cells that is neither iNOS^+^ nor CD163^+^.

### Condition 2: comparing pre-cross-Tx biopsies versus post-cross-Tx grafts

Our second aim was to compare the injury, renal endothelial (peritubular and glomerular) capillary rarefaction and general inflammatory status in post-Tx grafts versus pre-Tx (reference kidney). In HD-CR kidneys, GS and TI generally became more severe, glomerular endothelium and peritubular capillaries were lost, there were more glomerular ED1^+^ cells and a larger glomerular area. The importance of the recipient environment was confirmed by the association of preserved glomerular endothelium and decreased inflammation in the CKD graft (CD-HR versus CD-CR). Endothelial damage can originate from systemic and/or local factors. The association between oxidative stress, inflammation and CKD is well established (Tucker et al., 2015). Although in humans, endothelial function improves after successful renal transplantation (Kensinger et al., 2016), renal transplant recipients still have poor endothelial function in comparison to the healthy population (Stadler et al., 2009). Immunosuppressant drugs undoubtedly contribute to this effect (Ovuworie et al., 2001). As arterial pressure was similar in HD-CR versus HD-DR, the difference might be caused by the concomitant existence of oxidative and inflammatory systemic injury in CKD recipients (Rubattu et al., 2013).

We also observed that the CKD kidneys transplanted into CKD recipients do not appear to be additionally damaged as shown by no post- versus pre-Tx difference in the CD-CR group. Vercauteren et al. found that the remnant kidney is less susceptible to ischemia-reperfusion injury (IRI) in comparison to a healthy kidney (Vercauteren et al., 1999). In a subsequent study, the same group reported that a CKD environment causes resistance to IRI, and that the regeneration capacity of the transplanted healthy kidney, as assessed 10 days after transplantation, was not hampered by chronic uremia (Vercauteren et al., 2003).

Proteinuria is an important marker for kidney dysfunction. Patients with moderately reduced GFR without proteinuria have better clinical outcomes compared with patients with heavy proteinuria without abnormal GFR (Hemmelgarn et al., 2010). Loss of glomerular endothelium could be responsible for proteinuria (Obeidat et al., 2012; Salmon and Satchell, 2012). However, our results do not support this: healthy kidneys transplanted in CKD recipients had increased loss of glomerular endothelium but normal low levels of proteinuria (HD-CR, 12 mg/d; HD-HR, 11 mg/d). This means that endothelial damage could be less important for the development of proteinuria within the follow-up period of 6 weeks. Our results therefore suggest that in this model of transplantation, proteinuria might be driven by blood pressure. We observed a low level of proteinuria together with normal blood pressure and normal GFR in the HD-CR group, despite an increase in TI and GS.

### Condition 3: exploring the effects of recipient-derived cells in cross-Tx

The present cross-Tx study was also designed to address whether recipient bone-marrow health is important for graft function and structure. However, using eGFP^+^ recipients, we found no recipient effects on graft function or renal damage (see Condition 1). Preservation of glomerular endothelium was not associated with a difference in eGFP^+^ cell number, increases in cell proliferation, or differences in glomerular endothelial proliferation (JG12/Ki67). This suggests that the incorporation and proliferation of (circulating recipient-derived) cells is not a major factor in preservation and regeneration of glomerular endothelium in CKD. This is consistent with previous observations that renal artery injection of bone marrow cells protected against glomerular damage in experimental CKD, but was not related to incorporation or transdifferentiation of these cells into glomerular endothelium (Schirutschke et al., 2013; van Koppen et al., 2012a). Similarly, no differences were observed in the intensity of tubulo-interstitial eGFP^+^ staining. We found donor-dependent proliferation of peritubular endothelium; CD versus HD increased proliferation in the CR.

Regarding recipient-derived eGFP^+^/ED1^+^ cells, tubular and glomerular compartments seemed to be differently regulated; recipient environment determined the number of eGFP^+^/ED1^+^ cells in tubular fields, while in glomeruli, this was influenced by an interaction of donor and recipient effects. It has been previously demonstrated that, in tubular fields, the recipient environment determined MQ response to injury (Rowshani and Vereyken, 2012). In both tubular fields and glomeruli, a similar proportion of eGFP^+^ cells was found to be ED1^+^. Surprisingly, we found that most ED1^+^ cells were donor derived. The transplantation procedure and IRI-related response thus seemed to result in a relatively low influx of recipient inflammatory cells in donor kidneys. The remaining fraction of eGFP^+^ cells is probably positive for CD3 (also known as CD3E) (T-cell), CD20 (also known as MS4A1) (B-cell) or CD209 (also known as CD209A) (dendritic cell) (Bräsen et al., 2017).

Our study has some limitations. First, we did not perform time- and transplant-matched control testing for effects of the recipient environment. This is due to the chosen model of orthotopic left kidney transplantation: the same procedure for the right kidney is technically impossible. Transplanting two kidneys from one donor into two recipients would have required two microsurgeons to avoid systematically introducing differences in ischemia time between the first and the second kidney. However, to standardize ischemia times, all transplantations were performed by the same microsurgeon (D.A.P.). Second, the difference in median age between donors and recipients and the large variation in the ranges within the groups could be confounders in interpreting the results. These differences arose for logistic reasons because of the duration of the study. It should, however, be noted that there was practically full overlap in these ranges.

Our study provides a first glimpse into the complex regulation contributing to Tx outcome. Donor, recipient and donor-recipient interaction effects all play important roles in determining the status of renal peritubular and glomerular endothelium and inflammation. Implications of several of our findings regarding dynamics of inflammation and glomerular capillary rarefaction warrant further study in suboptimal versus optimal kidney grafts. A second focus could be on strategies to improve graft quality by *in vivo* or *ex vivo* treatment prior to transplantation surgery, and recipient cardiovascular status prior to and after transplantation surgery. In the long run, this will help with donor/recipient selection decisions as well as open avenues for novel therapeutic developments.

In summary, our study stresses the importance of the recipient environment, oxidative stress, endothelial proliferation, inflammation and vascular injury for graft outcome in renal transplantation, even when hypertension abates because of an initially healthy renal graft. It also raises the question of how to improve the recipient environment nonhemodynamically, to maintain long-term renal graft function and structure.

## MATERIALS AND METHODS

### Animals

The study protocol was approved by the Utrecht University Committee on Animal Experiments (DEC number 2012.II.03.053) and conformed with Dutch Law on Laboratory Animal Experiments. Male inbred Lewis rats (Charles River, Sulzfeld, Germany) were used in a preparatory study (*n*=21). In the transplantation study, Lewis males were used as donors (*n*=24) and eGFP^+^ Lewis males (*n*=24; GG2861Uex rats, own breeding colony) as recipients. Rats were housed in a climate-controlled facility with a 12:12-h light: dark cycle under standard conditions.

### Experimental design and groups

The following groups were used: HD-HR, healthy (H) Lewis donor (D) and healthy (H) eGFP^+^ recipient (R); CD-HR, CKD (C) Lewis donor (D) and healthy (H) eGFP^+^ recipient (R); HD-CR, healthy (H) Lewis donor (D) and CKD (C) eGFP^+^ recipient (R); CD-CR, CKD (C) Lewis donor (D) and CKD (C) eGFP^+^ recipient (R) (*n*=6/group). The cross-Tx design is depicted in Fig. 1. Body weights and ages of all rats at relevant stages of the experiment are shown in Tables S1 and S2.

### Models of CKD in rats

In a preparatory study (*n*=21 rats), we investigated whether injury was symmetrical in our bilateral model of CKD. Male Lewis rats (8 weeks of age) underwent two-thirds ablation of each kidney in a one-step procedure (BA); controls underwent bilateral sham surgery. Under isoflurane anesthesia, a median laparotomy was performed; arterial branches of both kidneys were carefully isolated under a Leica operation microscope and branches were sequentially coagulated, leaving only one branch/kidney intact, which supplied approximately one-third of that kidney. The area of ischemia was macroscopically checked on both the ventral and dorsal sides of each kidney to ensure that two-thirds of the renal mass of each kidney was infarcted (Papazova et al., 2014). SBP, urea and proteinuria were measured regularly. A terminal measurement, performed when proteinuria exceeded 100 mg/24 h in BA rats, included split-urine collection for single kidney function [GFR (inulin) and RPF (para-aminohippuric acid, PAH)] in left and right kidney (LK and RK). The femoral artery was cannulated in order to obtain direct measurement of mean arterial pressure (MAP) and a Transonic flow probe was placed on the left renal artery to measure renal blood flow (RBF). GS and TI were scored on PAS-stained 3 μm slides, previously embedded in paraffin. Control rats were age matched and sham operated.

LK-GS and LK-TI correlated strongly with RK-GS and RK-TI (r=0.79, *P*<0.001; r=0.89, *P*<0.001), respectively (Fig. S6, using correlation and Bland-Altman analyses). Proteinuria, in comparison with BP and urea, was the best predictor for GFR, RPF, TI and GS (r=−0.72, r=−0.63, r=0.81 and r=0.82, respectively, all *P*<0.001; Fig. S7). In this pilot study, bilateral ablation led to CKD, renal damage (GS and TI) was symmetrical and the best systemic predictor for function and injury was proteinuria. These findings firmly position this new bilateral ablation model in the field of transplantation.

To subsequently develop CKD in this strain for transplantation purposes, rats underwent two-thirds BA. Starting 1 week after surgery, development of CKD was accelerated with N(omega)-nitro-L-arginine (L-NNA), a NO-synthase inhibitor (200 mg/l) in drinking water (van Koppen et al., 2012a) and animals were fed standard powdered chow [CRM (E) FG; Special Diet Services Ltd., Witham, Essex, UK], with phosphate content of 0.63% and NaCl content of 0.74%, supplemented with 6% NaCl. After reaching proteinuria of 200 mg/d (6-9 weeks), L-NNA was withdrawn and salt supplement was removed from the chow 2 weeks later. As described previously (Kang et al., 2002), we also observed in pilot experiments that L-NNA and salt withdrawal resulted in an immediate systolic blood pressure and proteinuria decrease, which subsequently slowly recovered. CKD animals were included in the transplantation protocol when proteinuria levels reached a median of 111 mg/d (range 52-164) (without L-NNA and without salt) at a median age of 35 weeks (range 22-56). The experimental design is shown schematically in Fig. S8. At this stage, besides proteinuria, ablated rats demonstrate hypertension, high urea and a decrease in RBF, measured directly with a Transonic flow probe (Racasan et al., 2003). Healthy rats with intact kidneys were used as controls at a median age of 25 weeks (range 20-42).

We used injury in the right kidney as representative of injury in the left kidney at the time of transplantation (the isograft, post-Tx versus pre-Tx, depicted with asterisks in figures). Because proteinuria proved to be the best predictor of kidney damage in our preparatory study, all BA rats were screened and, where appropriate, matched with recipients based on proteinuria before entering the transplantation protocol.

### Kidney transplantation

We used orthotopic left kidney transplantation as described (Smit-van Oosten et al., 2001; Papazova et al., 2015), with subsequent removal of the right native kidney 10-14 days after transplantation.

For the donor procedure, donor rats were placed on an operating table with a heating pad keeping the body temperature at 37°C. A long abdominal incision was made from the sternum to the symphysis. The bowel was moved slightly to the left side, covered with moist gauze. The renal vessels and ureter were dissected carefully using atraumatic technique. Heparin was administered directly into the spleen, and 5 min after the left kidney was perfused with saline, the renal vessels were cut close to their junction to the aorta and vena cava and the ureter, ∼2 cm distal to the kidney hilum. Finally, the donor kidney was kept on ice with a standardized cold ischemia time of 30 min. All isografts were perfused and placed in organ-preserving solution Viaspan (Bristol Meyers Squibb, Hoofddorp, Netherlands) prior to transplantation. The right kidney of the donor rat was preserved in formaldehyde and then embedded in paraffin to evaluate pre-Tx histology (see above).

For the recipient procedure, the recipient rats were prepared in the same way as the donor rats except that heparin was not administered. The renal vessels were clamped using separate microvascular clamps and, including the ureter, were cut close to the kidney hilum. The donor kidney was placed and the following anastomoses were performed: end-to-end arterial anastomosis using eight to ten separate stitches; end-to-end venous anastomosis using continuous suture; end-to-end anastomosis of the ureter with four separate stitches. All anastomoses were performed with 10-0 prolene sutures. After completing the anastomoses at a standardized time of warm ischemia of 30 min, the microvascular clamps were released. The immediate patency of anastomosis was checked 20 min after clamp removal. After 10-14 days, the animals were anaesthetized again, the contralateral kidney was removed, the graft vessels were checked for late patency and long-term complications (infection, aneurysms), and the graft ureter anastomosis was checked for hydronephrosis. Only one case of hydronephrosis was observed, and this rat was excluded from the study.

### Longitudinal measurements

We performed tail-cuff SBP registration and collected 24-h urine samples for determination of protein excretion (Bradford Protein Assay, Bio-Rad Laboratories, Hercules, CA, USA) with the rats in individual metabolic cages while fasting, as described (Bongartz et al., 2010), at weeks 3 and 5 after transplantation. Blood samples were collected from the tail vein at the same time-points for determination of plasma urea (DiaSys Urea CT FS, DiaSys Diagnostic Systems, Holzheim, Germany).

### Terminal protocol

Terminal measurements were performed 6 weeks after Tx because one rat from the CD-CR group had to be euthanized as it reached the humane endpoint. Renal function was investigated under isoflurane anesthesia (Abbott Laboratories, Hoofddorp, Netherlands) as described (Racasan et al., 2003). MAP, GFR (inulin clearance), RPF (PAH clearance), and excretion of sodium and potassium (flame photometry) were measured. FE_Na_ and FE_K_ were calculated using standard formulae. At the end of the terminal protocol, rats were sacrificed by exsanguination via the aorta, perfused with 0.9% NaCl via the aorta at a perfusion pressure 10 mmHg above terminal MAP, and tissues were collected. Organ weights were noted. Furthermore, kidney samples (post-Tx) were fixed in 4% paraformaldehyde for embedding in paraffin, or snap-frozen for histological and immunohistochemical evaluation.

### Renal morphology and immunohistochemistry

TI and GS were scored on PAS-stained, paraffin-embedded slides (Joles et al., 2000, 1998). Scored variables for GS were matrix expansion, sclerosis, adhesion of Bowman's capsule and dilatation. GS was scored on 50 separate glomeruli by quadrants, on a scale of 0-4, where 0 means no quadrant was affected by any of these variables and 4 means that the whole glomerulus was affected. TI damage was scored on a scale of 1-5 in at least ten different nonoverlapping fields per rat. TI was defined as inflammatory cell infiltrates, tubular atrophy or interstitial fibrosis. All measurements were performed by an experienced technician or researcher blinded to the group allocation.

Peritubular and glomerular endothelial cells were stained with JG12 (mouse anti-JG12, BMS1104, 1:200; Bender Medsystems GmbH, Vienna, Austria). The endothelial (JG12^+^) area in at least ten tubular fields and 50 glomeruli per kidney was determined using Adobe Photoshop CS5 Extended, version 12.0×32 (Adobe Systems; San Jose, CA, USA). JG12^+^ area was corrected for glomerular area. ED1^+^ cells (pan monocyte/macrophage marker; mouse anti-rat CD68, ab31630, 1:250; Abcam, Cambridge, UK), iNOS^+^ cells (M1 MQ marker; mouse anti-rat NOS2, sc-7271, 1:100; Santa Cruz Biotechnology, Dallas, TX, USA) and CD163^+^ cells (M2 MQ marker; rabbit anti-rat CD163, ab182422, 1:500; Abcam) were counted in glomeruli (ED1, iNOS and CD163) and nonoverlapping tubular fields (ED1, CD163). TI score, GS, glomerular area, JG12 staining and ED1^+^ cell counts were performed in both donor (pre-Tx) and recipient (post-Tx) rats. Proliferative cells (Ki67 staining, rabbit anti-rat Ki67, RM-9106, 1:100; Thermo Fisher Scientific, Waltham, MA, USA) were counted in glomeruli of the recipient's graft kidney (post-Tx). Anti-GFP/RECA (rabbit anti-rat GFP, Ab6556, 1:200, Abcam; mouse anti-rat RECA, MCA970R, 1:100, Serotec, Oxford, UK) double staining was performed on 5 µm snap-frozen sections in the recipient's graft kidney, post-Tx, as described (van Koppen et al., 2012a). eGFP^+^ cells were counted in glomeruli, whereas in tubular fields we counted eGFP^+^ pixels. Anti-eGFP/ED1 double staining (1:200 for both, mouse anti-rat CD68, ab31630, 1:250; Abcam) was used to count all recipient-derived (eGFP^+^/ED1^+^) and donor-derived (eGFP^−^/ED1^+^) MQs in post-Tx kidneys. JG12/Ki67 double staining [enhanced using TSA Plus Fluorescein (JG12, 1:200; FITC 1:50) and TSA Plus Tetramethylrhodamine (Ki67, 1:100; TRITC, 1:50), Perkin Elmer Life Sciences, Boston, MA, USA] was used to count proliferation in glomerular endothelium and peritubular capillaries (JG12^+^/Ki67^+^) in post-Tx kidneys. Analyses were performed manually or with ImageJ software, version1.46r (National Institutes of Health, Bethesda, MD, USA).

### Oxidative and vascular damage

TBARS excretion was measured (TBARS Assay Kit, Cayman Chemical, Ann Arbor, MI, USA) in urine collected prior to termination as described (Attia et al., 2003; Papazova et al., 2015, 2014). Thoracic aortas from all rats were collected at termination and subsequently snap frozen. We performed von Kossa staining to detect abnormal calcium deposits (Finch et al., 2013; Koleganova et al., 2009).

### Statistics

Data are presented as mean±s.d. Student's *t*-test, two-way ANOVA or two-way repeated measures ANOVA, and Newman-Keuls post hoc test were used where indicated. The two-way analysis allows us to determine donor effects (CD versus HD, shown as ‘D’ in figures), irrespective of the recipient and vice versa recipient effects (CR versus HR, shown as ‘R’ in figures), irrespective of the donor, as well as interacting effects of the donor and recipient on graft function and structure. The two-way repeated measures analysis allows us to compare the time-dependent changes in the graft using the contralateral donor kidney as baseline-biopsy (post-Tx versus pre-Tx), time-dependent changes in the recipient for functional variables, and finally interaction between graft and recipient on such time-dependent variables.

## Supplementary Material

Supplementary information
