# Supplementary material for: Dissecting recipient from donor contribution in experimental kidney transplantation: focus on endothelial proliferation and inflammation
Source: Dis Model Mech. 2018 Jul 17;11(7):dmm035030. doi: 10.1242/dmm.035030 (PMC6078404; doi:10.1242/dmm.035030)
Supplement: Supplementary information [file dmm-11-035030-s1.pdf]

FIRST PERSON

First person – Diana Papazova

First Person is a series of interviews with the first authors of a selection of papers published in Disease Models & Mechanisms, helping early-career researchers promote themselves alongside their papers. Diana Papazova is first author on 'Dissecting recipient from donor contribution in experimental kidney transplantation: focus on endothelial proliferation and inflammation', published in DMM. Diana conducted the research in this article while studying for her PhD at the University Medical Center Utrecht, The Netherlands, with Prof. Dr Marianne Verhaar and Dr Jaap Joles. She is now a resident in anesthesiology at Amsterdam University Medical Centers, where her research interests include transplantation pathophysiology, renal oxygen handling and medical education.

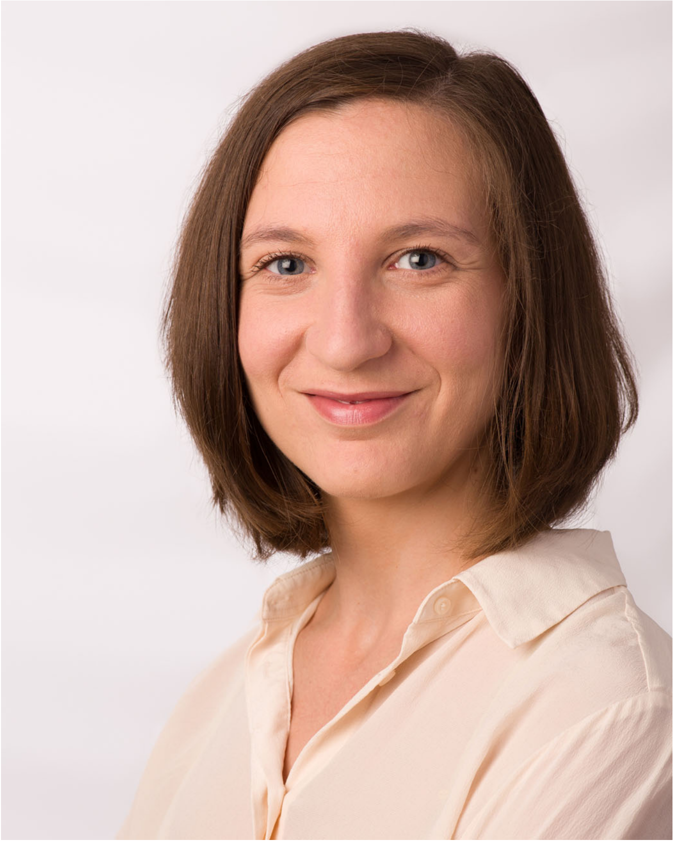

Diana Papazova. Photo credit: Jurrian Hartveldt.

How would you explain the main findings of your paper to non-scientific family and friends?

We were interested in how the donor and the recipient influence a kidney graft after kidney transplantation, its function and structure. First, we developed a model of chronic kidney disease in the rat, then performed kidney transplantations between healthy and diseased donors and healthy and diseased recipients in all possible combinations to dissect donor–recipient interactions. We showed that the function of the kidney graft after transplantation is solely dependent on the donor. However, oxidative and extra-renal vascular damage were determined by the recipient.

What are the potential implications of these results for your field of research?

Next to developing new strategies to optimize suboptimal kidney grafts prior to transplantation, we should also focus on strategies to improve the cardiovascular status of the recipients.

> "Having a chronic kidney disease model in the rat with two kidneys *in situ* and performing kidney transplantation using this model is unique."

What are the main advantages and drawbacks of the model system you have used as it relates to the disease you are investigating?

Having a chronic kidney disease model in the rat with two kidneys *in situ* and performing kidney transplantation using this model is unique. We used GFP^+ recipients from our own breeding program so we could track the origin of the cells. The only drawback is that creating this model is very time-consuming and it requires vigorous microsurgical training.

What has surprised you the most while conducting your research?

When transplanting a healthy kidney in a rat with chronic kidney disease (which resembles the clinical situation), we noticed that the kidney grafts had less glomerular endothelium compared to the grafts transplanted to a healthy control. Although this can be due to confounding factors I am curious to know to what extent the recipient environment can influence the quality of the kidney graft.

Describe what you think is the most significant challenge impacting your research at this time and how will this be addressed over the next 10 years?

Developing less time-consuming *in vivo* models to study chronic diseases. I do hope to see more innovation in this direction, including models other than rodents.

What changes do you think could improve the professional lives of early-career scientists?

> "[…] more funding that supports high-risk but potentially high-gain research ideas of young scientists is needed."

What most PhD students in basic sciences miss is a platform to show and publish their pilot studies. I believe this will make them more competitive when applying for grants. Also more funding that supports high-risk but potentially high-gain research ideas of young scientists is needed.

Diana Papazova's contact details: Amsterdam UMC, Location VUmc, Postbus 7057, 1007 MB Amsterdam, The Netherlands.

E-mail: d.a.papazova@gmail.com

Disease Models & Mechanisms (2018): doi:10.1242/dmm.035030: Supplementary information

What's next for you?

In this experimental setting we also looked at the cardiorenal axis and I look forward to showing these data. And for me personally, I am now focusing on my training to become an anaesthesiologist and combine clinical work and research.

Reference

Papazova, D. A., Krebber, M. M., Oosterhuis, N. R., Gremmels, H., van Zuilen, A. D., Joles, J. A. and Verhaar, M. C. (2018). Dissecting recipient from donor contribution in experimental kidney transplantation: focus on endothelial proliferation and inflammation. *Dis. Model. Mech.* **11**: dmm035030.
